# Vaccine candidate discovery for the next generation of malaria vaccines

**DOI:** 10.1111/imm.12780

**Published:** 2017-07-24

**Authors:** James Tuju, Gathoni Kamuyu, Linda M. Murungi, Faith H. A. Osier

**Affiliations:** ^1^ KEMRI‐Wellcome Trust Research Programme Centre for Geographic Medicine Coast Kilifi Kenya; ^2^ Department of Biochemistry Pwani University Kilifi Kenya; ^3^ Centre for Infectious Diseases Heidelberg University Hospital Heidelberg Germany; ^4^ Department of Biomedical Sciences Pwani University Kilifi Kenya

**Keywords:** antibodies, bioinformatics, *Plasmodium falciparum*, vaccines

## Abstract

Although epidemiological observations, IgG passive transfer studies and experimental infections in humans all support the feasibility of developing highly effective malaria vaccines, the precise antigens that induce protective immunity remain uncertain. Here, we review the methodologies applied to vaccine candidate discovery for *Plasmodium falciparum* malaria from the pre‐ to post‐genomic era. Probing of genomic and cDNA libraries with antibodies of defined specificities or functional activity predominated the former, whereas reverse vaccinology encompassing high throughput *in silico* analyses of genomic, transcriptomic or proteomic parasite data sets is the mainstay of the latter. Antibody‐guided vaccine design spanned both eras but currently benefits from technological advances facilitating high‐throughput screening and downstream applications. We make the case that although we have exponentially increased our ability to identify numerous potential vaccine candidates in a relatively short space of time, a significant bottleneck remains in their validation and prioritization for evaluation in clinical trials. Longitudinal cohort studies provide supportive evidence but results are often conflicting between studies. Demonstration of antigen‐specific antibody function is valuable but the relative importance of one mechanism over another with regards to protection remains undetermined. Animal models offer useful insights but may not accurately reflect human disease. Challenge studies in humans are preferable but prohibitively expensive. In the absence of reliable correlates of protection, suitable animal models or a better understanding of the mechanisms underlying protective immunity in humans, vaccine candidate discovery *per se* may not be sufficient to provide the paradigm shift necessary to develop the next generation of highly effective subunit malaria vaccines.

**Abbreviations**
mAbs: monoclonal antibodies
MSP: merozoite surface protein
PEXEL: *Plasmodium* export element
RAP: rhoptry‐associated protein

## Introduction

The need for a highly effective malaria vaccine remains urgent. The World Health Organization estimated that malaria afflicted over 200 million individuals across the globe in 2015, leading to approximately 429 000 deaths (World Malaria Report, 2016).^1^ Mortality was highest in young children in sub‐Saharan Africa. Although significant successes in malaria control have been realized through the scale‐up of preventive interventions such as insecticide‐treated bed nets, vector management and more effective treatment with artemisinin‐based combination drug therapy, these gains are threatened by the emergence of drug resistance.^2^, ^3^, ^4^ Coupled to this is the fact that if not well managed, successful control programmes can lay the foundation for massive resurgence.^5^ Many experts agree that for many parts of Africa the goal of malaria elimination will require the addition of effective vaccines to the malaria control tool box.^6^

### Current state of malaria vaccine development

**Figure 1:**
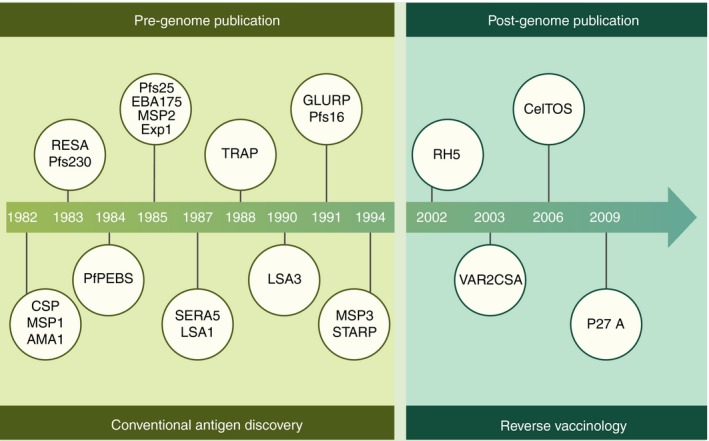
Historical timeline of vaccine candidate discovery for antigens under evaluation in clinical trials. Only 22 of the > 5400 proteins encoded in the *Plasmodium falciparum* genome are under evaluation in clinical trials. The majority of these were discovered in the pre‐genomic era. As illustrated for circumsporozoite protein, multiple trials for the same antigen have been conducted using different platforms and adjuvants, and in combination with a small number of other well‐studied parasite antigens. Adapted from the WHO Rainbow Tables http://www.who.int/immunization/research/development/Rainbow_tables/en/.^44^, ^45^, ^46^, ^54^, ^56^, ^58^, ^59^, ^61^, ^105^, ^106^, ^107^, ^108^, ^109^, ^110^, ^111^, ^112^, ^113^, ^114^, ^115^, ^116^, ^117^

Despite more than a century of extensive research, only one malaria vaccine candidate (RTS,S/AS01) has approval for use in countries where malaria is endemic.^7^, ^8^ Although this marks a major milestone in the history of malaria vaccine development, much more remains to be done. The vaccine had limited efficacy, which waned with time.^9^ Further evaluation to test the feasibility of its deployment alongside routine vaccinations in the expanded programme of immunization was recommended by the World Health Organization's expert committees. The RTS,S vaccine is based on a fragment of the circumsporozoite protein, one of the > 5400 proteins encoded in the parasite's genome.^10^ Altogether, only 22 parasite proteins have been under evaluation as sub‐unit vaccines in clinical trials (Fig. 1). In many cases, the same vaccine candidates have been tested repeatedly, albeit on different platforms, with different adjuvants and increasingly, in combination with a small number of other well‐studied parasite antigens (Table 1).

**Table 1:** Vaccine constructs containing circumsporozoite protein (CSP) that have been tested in clinical trials

RTS,S/AS01E
RTS,S‐AS01 delayed fractional third dose
ChAd63/MVA ME‐TRAP^a^
ChAd63/MVA ME‐TRAP + Matrix M™^a^
CSVAC
R21/AS01B
R21/Matrix‐M1
R21 adjuv (RTS,S‐biosimilar) with ME‐TRAP combined
NMRC‐M3V‐Ad‐PfCA^a^
NMRC‐M3V‐D/Ad‐PfCA Prime/Boost^a^
RTS, S/AS02A
DNA/MVA CSP
FP9 CSP + LSA‐1 epitope/MVA CSP + LSA‐1 epitope^a^
DNA/MVA prime‐boost Multi‐Epitope (ME) string + TRAP^a^
FP9 MVA prime‐boost ME‐TRAP^a^
HepB Core‐Ag CSP‐VLP
RTS,S/AS02 and FMP1/AS02^a^
RTS,S/AS02 and SSP2/TRAP^a^
RTS,S/AS02 and MVA CSP
RTS,S/AS02 and DNA CSP
CSP DNA immunization
MuStDO5 (Multi‐Stage DNA vaccine Operation, five antigens)^a^
NMRC‐MV‐Ad‐PfC
CSP long synthetic peptide
Adenovirus (Ad26) vectored CS; Adenovirus (Ad35) vectored CS
Adenovirus (Ad35) vectored CS
Adenovirus (Ad35) and adenovirus 26 (Ad26) vectored CS in heterologous prime‐boost regimen
ChAd63/MVA (monovalent CS or ME‐TRAP) in prime boost regimen
ChAd63/MVA (multivalent CS, ME‐TRAP or apical membrane antigen 1) in prime boost regimen^a^
Adenovirus (Ad35) vectored CS and RTS.S‐AS01 in heterologous prime‐boost regimen

a Combination vaccines containing CSP. Adapted from the WHO Rainbow tables http://www.who.int/immunization/research/development/Rainbow_tables/en/.

### Key challenges

A fundamental issue hindering the development of highly effective malaria vaccines is the lack of reliable or reproducible correlates of protection. Passive transfer studies provided strong evidence that antibodies are key components of acquired immunity against malaria.^11^ However, although antibodies against many different parasite antigen(s) have been proposed as correlates of immunity, the findings are often inconsistent between studies^12^ and none have been universally accepted. Although a range of methodological issues contribute to this lack of clarity, equally important has been the difficulty in defining ‘who is protected from malaria’ in cohort studies.^13^ Hence, it remains unclear which of > 5400 proteins encoded in the parasite genome induce protective antibodies. Even when potential vaccine antigen(s) are identified, overcoming parasite diversity (many of the vaccine candidates are polymorphic), redundancy (for example in receptor–ligand interactions required for red blood cell invasion) and the induction of long‐term immunity remain major challenges.

### Vaccine candidate discovery

Antigen discovery for malaria vaccine development can be broadly considered in terms of: (i) the pre‐genomic era, marked by the publication of the *Plasmodium falciparum* genome^10^ and (ii) the post‐genomic era. A review of the vaccine candidates currently under clinical development reveals that approximately 82% (18/22) were discovered in the pre‐genomic era, largely through the screening of genomic and cDNA libraries for antibodies with defined specificities or functional activity. Antigen discovery (the commonly used term) was typically slow and a single or handful of antigens was reported in any given publication (Fig. 1). Currently the availability of genomic, transcriptomic and proteomic data sets reviewed by Proietti *et al*. in ref. 14 provide an unparalleled opportunity for the systematic interrogation of the entire parasite genome to identify potential targets of protective immunity at scale. The term ‘antigen discovery’ is now potentially a misnomer as genomes have been annotated and practically all antigens in effect are ‘known’. We review what we now term ‘vaccine candidate discovery’ for sub‐unit *P. falciparum* vaccines in the post‐genomic era and argue that although we have exponentially increased our ability to rapidly identify multiple potential candidates simultaneously, a significant bottleneck remains in their validation and prioritization for evaluation in clinical trials.

## Reverse vaccinology

**Figure 2:**
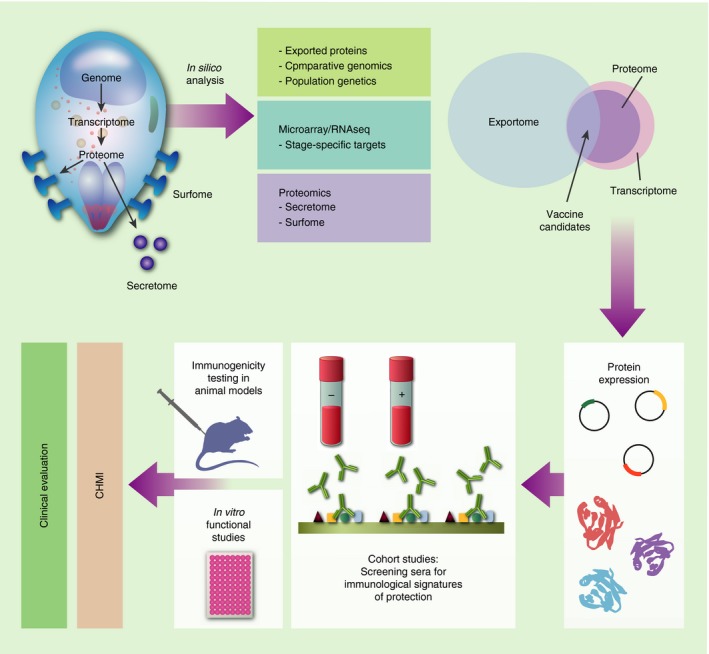
Reverse vaccinology 1·0. In the post‐genomic era, discovery of vaccine candidates is increasingly reliant on an integrated analysis of the genomic, transcriptomic and proteomic data sets. The targets that are prioritized through this approach are subsequently validated in the laboratory using *in vitro* assays, cohort studies and animal models before testing for immunogenicity and efficacy in humans.

In reverse vaccinology high‐throughput *in silico* analyses of genomic, transcriptomic or proteomic parasite data sets are applied to identify features that might distinguish potential vaccine candidates^15^ (Fig. 2). These could include markers of cell surface expression such as glycosylphosphatidylinositol anchors, signal peptides, transmembrane domains or of extracellular protein secretion such as the *Plasmodium* export element (PEXEL) motif.^16^, ^17^ A genome‐wide *P. falciparum* screen for signal sequences and/or the PEXEL motif defined an ‘exportome’ comprised of 396 genes. Subsequent comparative genomics in multiple *Plasmodium* species identified a novel PHIST (*Plasmodium* helical interspersed subtelomeric) protein family that was greatly expanded in *P. falciparum* 18 and may be of interest for vaccine development. In functional comparative genomics the function of a set of genes in one species is characterized that might then be applicable to its homologues. For example, the P48/45 gene family members are expressed on the surface of gametocytes and conserved in *P. falciparum*,* P. vivax*,* Plasmodium berghei* and *Plasmodium yoelii*.^19^ Disruption of the *P48/45* in *P. berghei* demonstrated its role in fertility of the male gamete making its homologue in *P. falciparum* a potential transmission‐blocking vaccine candidate.^20^

Population genetic analyses aim to detect polymorphic genes that appear to be under selective pressure, for example as a result of host immunity. Allele‐specific antibodies acting on polymorphic parasite targets are thought to exert negative frequency‐dependent selection on a major circulating allele while allowing the minor allele to increase within the population.^21^ This culminates in balancing selection on important immune targets, which maintains allelic variants at intermediate frequencies that are higher than would be explained by other mechanisms, such as genetic drift, alone. Signatures of balancing selection can therefore be used to identify potential immune targets as was recently the case for MSPDBL2,^22^, ^23^ which then need subsequent validation.^24^ Although attractive, overcoming parasite diversity at important loci that are polymorphic remains a significant challenge for vaccine development.^25^

Transcriptomics provides a more targeted approach to antigen identification through the quantification of stage‐specific mRNA transcripts.^26^ In early studies, microarray chips were pre‐loaded with DNA probes designed to hybridize to fluorescently labelled cDNA generated from mRNA.^27^, ^28^ More recently, RNA sequencing (RNA‐seq) is considered the better option as cDNA is obtained through next‐generation sequencing methods that do not require prior knowledge of the genome sequence and enable the detection of polymorphic genes.^29^ Additional advantages include the ability to detect alternative splice variants and more accurate quantification of RNA expression on account of a wider dynamic range. Multiple RNA‐seq data sets are now available for *Plasmodium*.^14^ Transcriptomic analyses have been coupled to experiments in animal models for vaccine candidate discovery. In a proof‐of‐principle genetic immunization study 19 exons were selected from the *P. yoelii* genome based on their transcription in sporozoites and directly used as DNA vaccines in mice. The PY01316 antigen was found to be associated with a 95% reduction of hepatic parasites compared with unvaccinated controls and has an orthologue in *P. vivax*, making it a potential vaccine candidate.^30^

In structural bioinformatics the three‐dimensional conformational structures of proteins are deduced from sequence data and can guide the design of epitope‐based vaccines. In a principle to practice example, Villard *et al*. scanned the *P. falciparum* genome for proteins predicted to be extracellular and containing the conserved *α*‐helical coiled coil structural motifs that predicted epitope stability.^31^ Further analysis delineated regions within these selected proteins that had limited polymorphisms and a low probability of forming conformational epitopes. The peptide P27A emerged from this line of investigation and was widely recognized by sera from semi‐immune individuals; antibodies against it were associated with a reduced risk of malaria and it was formulated as a peptide vaccine for clinical trials (Table 1).^32^

It might be argued that *in silico* approaches introduce a degree of bias because *a priori* knowledge of ‘desirable characteristics’ is required to guide screening. A sound scientific basis for the determination of these characteristics mitigates against this bias and provides a rational route for vaccine candidate discovery. Importantly further validation *in vitro* and *in vivo* where possible, will always be required.

## Proteomics

Proteomics refers to the large‐scale identification and/or quantification of the set of proteins produced in the biological context either by whole organisms, organs or organelles. Proteomes are not constant; they differ from cell to cell, fluctuate over time and may respond differently in response to external stimuli. Multiple proteomic‐based experimental platforms have been applied to vaccine candidate discovery and predominantly focus on the identification of immunogenic or surface‐exposed, secreted or membrane proteins of pathogens. For example the Spy0416 and Spy0269 antigens of group A *Streptococcus* bacteria that are currently in pre‐clinical stages of evaluation were identified through proteomic analyses of bacterial membranes and subsequently validated using mouse models.^33^, ^34^

**Figure 3:**
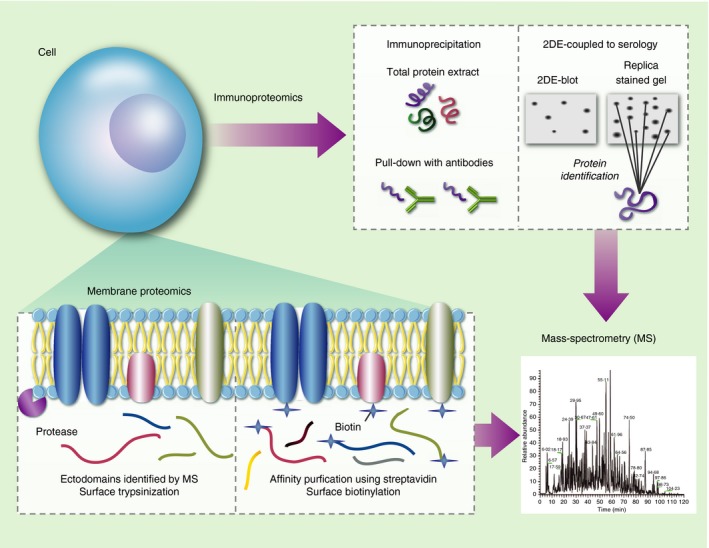
Proteomic‐based vaccine candidate discovery. This strategy identifies proteins that interact with the host immune system or that are localized to the surface of pathogens. Proteins bound to antibodies are either immune‐precipitated directly from complex mixtures or separated by two‐dimensional gel electrophoresis before probing with antibodies and identification by mass spectrometry. In surface trypsinization or surfomics, intact membranes are exposed to short‐term treatment with proteases resulting in the release of protein ectodomains. Surface proteins can also be selectively tagged with a cell‐membrane‐impermeable biotin reagent and affinity purified using streptavidin.

A range of experimental methods are applied to solubilize and separate individual proteins based on their biochemical characteristics before identification by mass spectrometry.^35^, ^36^ For *P. falciparum*, these have included two‐dimensional gel electrophoresis,^37^ membrane solubilization and purification,^38^ and surface biotinylation^39^, ^40^ (Fig. 3). Well‐studied vaccine candidates such as merozoite surface protein 1 (MSP1), MSP2, serine repeat antigen and EXP1 have been identified alongside long lists of secreted, surface‐associated or surface‐anchored parasite proteins,^37^, ^38^ some of which were subsequently identified as correlates of protection in cohort studies.^41^, ^42^ Additional proteins on the surface of infected erythrocytes^43^ and on sporozoites^39^, ^40^ have also been identified and may be potential vaccine candidates.

Immunoproteomics homes in on proteins that interact directly with the host's immune system such as protective antibodies. These immunoglobulins have been used in immunoprecipitation (pull‐down) experiments, to identify protein–protein interactions or coupled to two‐dimensional gel electrophoresis (Fig. 3). For example, monoclonal antibodies (mAbs) with *in vitro* parasite growth inhibitory activity or shown to interact with monocytes leading to parasite death were used to immunoprecipitate well‐characterized antigens such as apical membrane antigen 1, MSP1, MSP2 and MSP3.^44^, ^45^, ^46^, ^47^ More recently MSP6, MSP7 and MSP9, as well as the rhoptry proteins RhopH1, rhoptry‐associated protein 1 (RAP‐1) and RAP‐3, were identified as the interaction partners of MSP1 and RhopH3 in pull‐down experiments using merozoite lysates and the respective anti‐sera.^48^ Although the coupling of two‐dimensional gel electrophoresis to serology has been used successfully to identify vaccine candidates for bacterial infections such as *Neisseria meningitidis* 49 and *Brucella melitensis* 50 its use in *Plasmodium* appears to have been limited to only two studies. The first was reported before genomic data were available and involved the analysis of *Plasmodium knowlesi* schizont membrane proteins using serum from Rhesus monkeys,^51^ whereas in the second, membrane proteins from *P. falciparum* infected erythrocytes were probed using sera from malaria‐naive travellers.^52^ This may therefore represent a neglected but attractive approach to target discovery in *P. falciparum* that would exploit both the availability of genomic databases and well‐characterized human serum samples. For example, sera from individuals who achieved sterile protection following experimental controlled human malaria infection were probed against a *P. falciparum* library,^53^ and could similarly be used to analyse parasite proteins resolved by two‐dimensional gel electrophoresis.

Although proteomics can be more focused on distinct organelles, like other reverse vaccinology approaches it still results a formidable number of potential vaccine candidates that need further evaluation. The decision to prioritize a given protein or set of proteins over others remains challenging.

## Screening of cDNA expression libraries

For most genes mRNA acts as a good proxy of protein expression.^26^ Complementary DNA (cDNA) libraries can be obtained from the reverse transcription of the mRNA transcripts isolated from a specific parasite developmental stage. These can be cloned into appropriate vectors and expressed as recombinant antigens typically in bacteria or phages as individual clones, each expressing a single protein. The complete proteome can then be disentangled into individual components without any filtering bias, and immunoreactive proteins and their corresponding genes can be simultaneously identified. The success of this approach in vaccine antigen discovery relies critically on the careful selection of the sera used to probe the expression library. In the pre‐genomic era this approach contributed to the discovery of at least eight *P. falciparum* vaccine candidates that have been tested in clinical trials (Fig. 1 and refs 54, 55, 56, 57, 58, 59, 60, 61). It remains a powerful platform for screening potential vaccine antigens, as was recently demonstrated in the identification of *P. falciparum* schizont egress antigen.^62^ One disadvantage of this system is that it may introduce a bias by over‐representation of dominantly expressed transcripts. Another is that proteins are typically expressed in *Escherichia coli*, which often is not ideal for plasmodia.^63^

## Functional antibody‐guided vaccine candidate discovery

**Figure 4:**
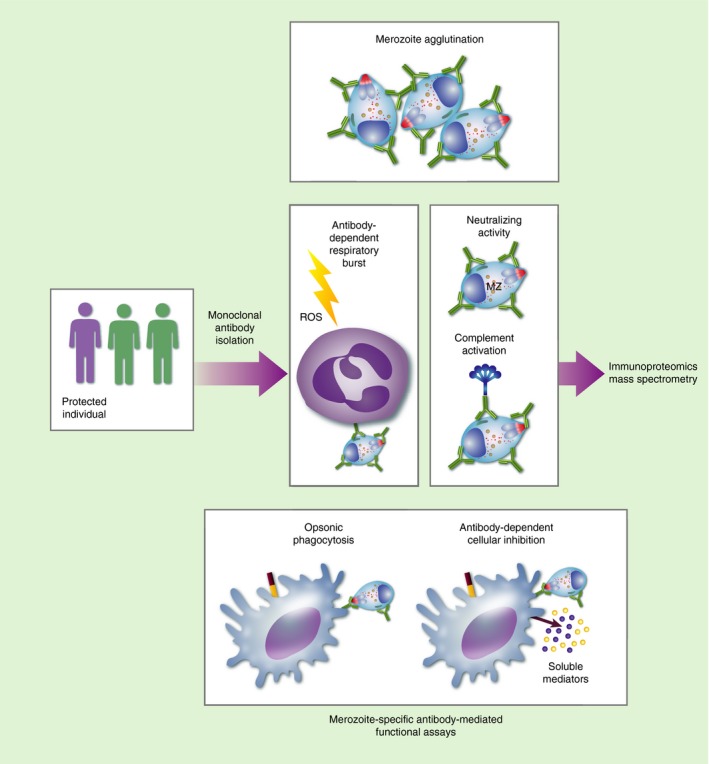
Functional antibody‐guided vaccine candidate discovery. Malaria immune donors are identified and human monoclonal antibodies are isolated from memory B cells or plasma cells. The monoclonal antibodies are screened for effector functions targeting stages of the parasite life cycle such as merozoites in assays of agglutination, neutralizing activity, complement activation, antibody‐dependent respiratory burst and antibody‐dependent cellular inhibition. Antigens or epitopes targeted by functional antibodies are identified by immunoproteomic approaches and mass spectrometry.

The methods described provide no guarantee that the potential vaccine candidates discovered in this way will induce immune responses that actually kill or inactivate parasites (possible mechanisms illustrated in Fig. 4). In what has been termed Reverse Vaccinology 2·0 by Rappuoli *et al*.,^64^ vaccine candidate discovery begins with an understanding of the characteristics of a successful antibody response and the subsequent identification of the target antigen that induces those antibodies. Mimotopes corresponding to respective mAbs can then be designed and tested as immunogens. For instance, a panel of mAbs that neutralize human cytomegalovirus infections in multiple cell types were isolated from infected donors and the important epitope was mapped to nine sites on the gH pentameric complex that is required for viral infection.^65^, ^66^ Previous vaccine development efforts had focused on glycoprotein B, which induced neutralizing antibodies that were less potent than those targeting the pentameric complex.^67^ Similarly a respiratory syncytial virus‐specific mAb (MPE8) that cross‐neutralizes paramyxoviruses was recently isolated and shown to bind to the pre‐fusion F glycoprotein;^68^ unlike palivizumab, a licensed therapeutic mAb that binds to the post‐fusion viral F protein. In HIV, several broadly neutralizing antibodies of high potency have been identified and target epitopes have been mapped to the highly variable envelope glycoprotein.

Although logically attractive, this approach has not been universally successful. The greatest successes so far have been achieved with viral infections for which a single and high‐throughput neutralization assay adequately predicts protection. A fundamental challenge in the application of this strategy to malaria is the lack of a reliable functional correlate of immunity. Although the responses measured in a number of assays predicted protection,^69^, ^70^, ^71^, ^72^, ^73^, ^74^ there is no consensus on which of these is the best, and the findings are often inconsistent between studies.^69^, ^70^, ^71^, ^75^ Recently, Tan *et al*. screened sera obtained from malaria‐immune adults for broad reactivity against variant surface antigens in a mixed agglutination assay and identified B cells secreting a pan‐reactive mAb that bound to 2 repetitive interspersed family proteins (RIFINs) on the surface of infected red blood cells.^76^ At least one prospective study found a protective association between the ability of sera to agglutinate red cells infected with parasites and severe malaria but this protection was largely variant specific.^77^ Another mAb obtained from immortalized memory B cells of women with pregnancy associated malaria bound to VAR2CSA,^78^ a specific variant surface antigen associated with placental infected *P. falciparum* erythrocytes that had already been prioritized for vaccine development and is currently under clinical evaluation (Table 1). Antibodies against VAR2CSA from multiparous pregnant women can inhibit the binding of infected erythrocytes to the placenta^79^ and this was associated with improved birth outcomes.^80^, ^81^, ^82^

A common theme that emerges from applying functional antibody‐guided vaccine candidate discovery to complex organisms is that important antibodies appear to form a minority or rare population in the overall immune response within individuals and are even less frequent at a population level. Screening for important mAbs is therefore technically challenging and although proof of principle has been achieved with viruses, it remains to be seen whether or not this strategy will work against more complex organisms. For instance, in HIV, none of the immunogens targeted by broadly neutralizing mAbs have elicited antibodies of similar breadth or potency following immunization in humans.^83^, ^84^ The design of immunogens that mimic the native epitopes identified by functional antibodies or those that activate the germline B‐cell precursors are currently being explored.^83^ We postulate that for complex pathogens like *Plasmodium*, what may be required are combinations of antibodies that target multiple epitopes or those that act in synergy to achieve a protective response.

## Antigen expression

To immunologically characterize and further validate potential vaccine candidates they need to be expressed and purified in recombinant form, ideally while maintaining their native conformation. Technical challenges in the expression of *P. falciparum* proteins in heterologous systems have hindered vaccine development.^85^, ^86^, ^87^ For example, an attempt at large‐scale expression of 1000 open reading frames from *P. falciparum* in *E. coli* yielded only 63 soluble proteins.^85^ We briefly highlight here three of the most widely employed systems for protein expression in *P. falciparum*. The *E. coli* expression system is probably the most attractive and the most widely used because it is relatively cheap, often yields high protein quantities, is relatively quick and does not require expensive laboratory equipment. Though attractive, proteins may be of poor quality, particularly with regard to proper folding, may form inclusion bodies and lack important post‐translational modifications.^88^

Cell Free expression systems use crude extracts from cells that do not contain cell walls to drive the transcription and translation of proteins from exogenous DNA. The crude extracts are prepared from cultured microbial, plant or animal cells and are usually supplemented with amino acids and high‐energy substrates. The absence of a cell wall/membrane provides an open environment that allows the direct translation of genes from linear DNA fragments without the need to clone them into vectors. In *Plasmodium* spp. this has been achieved using either an *E. coli* or wheatgerm cell‐free *in vitro* transcription and translation system.^89^, ^90^ *Escherichia coli* extracts are prepared from bacterial cell lysates whereas wheatgerm extracts are prepared from ground wheat embryos.^91^

Cell‐free protein synthesis systems are high throughput and rapid, bypassing the time‐consuming cloning of individual genes by synthesizing proteins directly from PCR products.^92^ A common disadvantage is that protein translation occurs in reducing environments that may compromise the formation of disulphide bonds that are important for the native conformation of extracellular proteins.

Mammalian cell lines share the parasites’ similar eukaryotic protein translational and post‐translational modifications that include proper folding, the ability to form disulphide bond linkages and secretion of synthesized proteins into the extracellular environment, which limits toxicity and accumulation of recombinant antigens in the cell. Unlike the two previous systems discussed above, large full‐length (open reading frames > 3 kb) and correctly folded proteins can be expressed using this system.^93^ However, it is comparatively low throughput and costly.

## Validation of potential vaccine candidates

Having ‘discovered’ and expressed a list of potential vaccine candidate antigens that may stretch from a manageable handful to several hundreds, the key question then becomes how to validate and prioritize these for the expensive journey of actual vaccine development. With a few exceptions, pre‐clinical trial candidate validation in malaria largely relies on traditional longitudinal cohort studies, functional bioassays, animal models ahead of clinical trials in humans. None of these platforms is perfect and we make the case that herein now lies one of the major bottlenecks to malaria vaccine development.

Longitudinal cohort studies are powerful tools used to examine exposures or risk factors against pre‐specified outcomes. Although causality is not established, factors associated with a reduced risk of a given disease are commonly referred to as being ‘protective’.^94^ In one of the earliest studies that applied this study design to malaria, sickle cell trait and bed‐net use were correctly identified as being protective and lent support to the approach, although the authors repeatedly urged cautious interpretation.^95^ Since then, although many antigens (including many of those currently in clinical trials) have been proposed as correlates of protection using this study design, the results are often inconsistent between studies.^12^ These disparities probably stem from the challenges of defining both the exposure (for example antibody measurement) and the end point (i.e. ‘who’ is protected, from what and for how long) reviewed by Marsh and Kinyanjui.^13^, ^96^ Neither the exposure nor the end point (and all the technical steps in between) have historically been standardized across studies. Coupled to this with regards to the human host is the diverse and often fluctuating intensity of malaria transmission, the diversity of genetic backgrounds and, increasingly, the more widespread use of interventions to prevent malaria such as insecticide‐treated bed nets.^97^ The antigenic diversity of multiple parasite proteins within the parasite and induction of allele‐specific immunity adds an extra layer of complexity.^98^ Against this backdrop, it is therefore not surprising that the evidence base for any given vaccine candidate from this type of validation is relatively weak.

Demonstration of antigen‐specific antibody function is often considered as additional supportive evidence in the validation of potential vaccine candidates. A key issue here is the limited understanding of the mechanisms that underlie protective immunity and the lack of a single assay that reliably predicts protection. As such, although antibodies against many antigens have been reported to inhibit parasite growth *in vitro*, this activity has not consistently correlated with protection.^73^ Many additional mechanisms have been proposed but few are widely used and methodologies are not standardized.^99^ It remains possible that one mechanism may be insufficient to account for immunity and in one study we observed a strong correlation with protection against severe malaria in infants and young children with both growth inhibitory activity and antibody‐dependent respiratory burst in neutrophils.^69^ The IgG‐dependent opsonic phagocytosis activity of monocytes against merozoites correlated strongly with protection in two recent independent studies.^70^, ^75^ We further demonstrated that affinity‐purified human antibodies against MSP2 and MSP3 that had previously been shown to be associated with a lower risk of clinical malaria in the same Kenyan population were effective in the opsonic phagocytosis activity, suggesting that this may be an important mechanism.^70^ Additional studies in varying contexts of malaria transmission intensity are needed, as well as studies to define the concentration of antigen‐specific antibodies that would be necessary *in vivo in* humans to overcome an infection and that could be realistically induced by vaccination.

Animal models of *Plasmodium* infection have an important role to play in the initial assessments of safety, immunogenicity and potential protective efficacy of vaccine candidates. Although positive results with regards to protection would be considered as supportive despite the differences between rodent and human malarias, negative ones do not necessarily exclude the likely importance of an antigen in the latter. Animal models can also facilitate the elucidation of the mechanisms important for protection, for example analyses of liver‐stage infections that cannot be undertaken in humans.^100^ The humanized mouse model in which mice can be infected with *P. falciparum* is increasingly used but best suited to passive transfer experiments following challenge with regards to vaccine candidate validation.^101^, ^102^ The use of great apes and monkeys as models is now increasingly ethically unpalatable and carried out in only a few specialized centres worldwide.

### Challenge studies in humans – the ultimate test?

Clinical trials in humans ultimately have to be done and can rapidly provide valuable insights on the immunogenicity, safety and potential efficacy of vaccine candidates, which could be tested and improved iteratively. This platform could also be a powerful tool for vaccine candidate discovery, comparing sera from individuals who are protected following challenge to those who are not under identical and well‐controlled experimental conditions.^53^, ^90^, ^103^ The misclassification bias inherent in traditional cohort studies is minimized as both the exposure (timing of infection, challenge dose) and outcome (development of blood stage parasitaemia) are standardized. On the other hand, infections are often monoclonal and may not accurately reflect the immune mechanisms induced by repeated polyclonal infections in areas of high malaria endemicity. In one such recent trial, although protection was achieved with a high dose of non‐irradiated sporozoites in adults on chloroquine chemoprophylaxis, an antigen‐specific correlate of protection was not identified.^104^

Scientific considerations aside, such trials are prohibitively expensive and require considerable infrastructure with regards to regulatory frameworks, human personnel and Good Clinical Laboratory Practice‐qualified laboratories. Pre‐clinical production of vaccines to Good Manufacturing Practice standards suitable for injection into humans, and for toxicology and immunogenicity testing are also incredibly expensive, require evidence‐based decisions on adjuvants, carriers and vaccination schedules, and must undergo lengthy international regulatory approvals. Hence, validation (Fig. 1) and/or discovery using this platform is restricted to a small but growing number of vaccine candidates and research centres and remains a major hurdle for vaccine development.

## Concluding remarks

Although the vaccine candidate discovery toolbox has increased considerably in the post‐genomic era, building sufficient evidence to warrant the long and expensive journey of clinical testing remains a significant bottleneck. Innovative studies that could help to accelerate the validation of potential vaccine candidates are urgently required to maximally use the wealth of data emerging from a wide range of vaccine candidate discovery platforms. Ultimately, expensive clinical trials have to be conducted and it is our view that without a better understanding of the mechanisms by which humans acquire immunity, and in the absence of serendipity, the development of sub‐unit vaccines against malaria will continue at a snail's pace.

## Disclosures

There are no competing interests.
